# Genotypic Diversity and Antimicrobial Resistance Profiles of Multidrug-Resistant *Escherichia coli* in Porcine Populations from Hubei, China

**DOI:** 10.3390/ijms27010524

**Published:** 2026-01-04

**Authors:** Xiaoyue Li, Zewen Liu, Ningning Wang, Rui Guo, Wenjie Chen, Wei Liu, Ting Gao, Keli Yang, Yongxiang Tian, Fangyan Yuan

**Affiliations:** Key Laboratory of Prevention and Control Agents for Animal Bacteriosis (Ministry of Agriculture and Rural Affairs), Hubei Provincial Key Laboratory of Animal Pathogenic Microbiology, Institute of Animal Husbandry and Veterinary, Hubei Academy of Agricultural Sciences, Wuhan 430064, China

**Keywords:** *Escherichia coli*, antibiotic resistance, MLST, extended-spectrum β-lactamase, ESBLs, resistance genes

## Abstract

The indiscriminate and excessive use of antimicrobial agents in livestock production is a major driver of antimicrobial resistance (AMR), thereby posing a grave threat to global public health. Although several surveillance studies have documented antimicrobial resistance patterns of swine-derived *E. coli* in different regions of China, comprehensive investigations integrating multilocus sequence typing (MLST), resistance determinants, and virulence gene profiles have remained scarce for central China, particularly Hubei province, since 2018. This study investigated the prevalence of antibiotic resistance, and molecular epidemiology of *E. coli* isolated from swine farms in Hubei province, China, while simultaneously analyzing their clonal and genetic diversity. A total of 148 *E. coli* isolates were collected from porcine sources in central China, revealing distinct regional variations in genetic diversity. Multilocus sequence typing (MLST) analysis identified 38 sequence types (STs) distributed across 7 clonal complexes (CCs) and several unassigned clones. ST46 emerged as the predominant sequence type (19.6% prevalence), followed by ST23 and ST10. Antimicrobial susceptibility testing demonstrated 100% resistance to lincosamides and sulfonamides, with all isolates exhibiting multidrug resistance (MDR) to antimicrobial classes. Genetic characterization detected 16 resistance determinants, with individual isolates carrying 5–7 resistance genes on average. The resistance profile included seven β-lactamase genes: blaTEM (61.5%), blaCTX-M-1G (57.4%), blaDHA (46.6%), blaSHV (39.2%), blaCTX-M-9G (24.3%), blaOXA (13.5%), and blaCMY-2 (1.4%); and eight aminoglycoside-modifying enzyme genes, including polymyxin resistance gene mcr-1 (7.4%). Virulence factor screening through PCR detected nine associated genes, with EAST1, fyuA, STa, K88, STb, Irp2, and LT-1 present in 95.3% of isolates, while K99 and 987P were absent in all specimens. This investigation documents alarmingly high antimicrobial resistance rates in swine-derived *E. coli* populations while elucidating their genetic diversity. The findings suggest that intensive antibiotic use in porcine production systems has driven the evolution of extensively drug-resistant bacterial isolates. These results emphasize the urgent need to implement antimicrobial stewardship programs in livestock management to mitigate AMR proliferation.

## 1. Introduction

*Escherichia coli* is a predominant commensal bacterium residing as part of the natural gut microbiota in humans and warm-blooded animals [1,2,3,4]. However, pathogenic isolates of *E. coli* are a major cause of intestinal infections in neonatal piglets aged 1–10 days, primarily causing diarrhea and edema in this vulnerable population [5]. The lack of effective vaccines to prevent *E. coli* outbreaks attributable to its diverse serotypes and region-specific epidemiological variations has led to a heavy reliance on antimicrobial agents for disease management in most agricultural settings. The escalating use of antimicrobials in livestock production has precipitated the emergence of multidrug-resistant (MDR), extensively drug-resistant (XDR), and even pandrug-resistant (PDR) bacterial isolates, raising significant global health concerns. Of particular urgency is the rapid dissemination of antimicrobial resistance (AMR), a critical public health crisis that now permeates multiple sectors of society and imposes substantial economic burdens worldwide [6].

Antimicrobial resistance (AMR) has evolved into one of the most critical global health threats of the past three decades [7]. Of particular concern is the rising prevalence of drug-resistant Gram-negative bacteria in livestock populations, which can transmit to humans through environmental pathways and food chain contamination [8]. The intensive use of antibiotics in swine production has exacerbated this trend, with resistance profiles in pig-derived pathogens showing accelerated development when compared with other livestock sectors. Notably, surveillance data from Great Britain revealed higher AMR prevalence in porcine-derived *E. coli* isolates than those from cattle and sheep [9], underscoring the urgency to systematically characterize antibiotic resistance profiles of pathogenic *E. coli* in Chinese swine operations. Surveillance data from 2011–2012 revealed high resistance rates in swine *E. coli* isolates to tetracycline (79.57%), trimethoprim-sulfamethoxazole (73.12%), and kanamycin (55.91%) [10]. Subsequent monitoring in Guangdong province (2013–2016) detected multidrug resistance (MDR) in 100% of 333 *E. coli* isolates from commercial pig operations [11]. A surveillance of *E. coli* in Chinese pig farms from 2018 to 2019 indicated that 90.54% were multidrug-resistant (MDR) or had common resistance to tetracycline (96.26%) etc., and occasional resistance to last-resort drugs. Among 515 sequenced MDR isolates were 101 O-serogroups, 118 sequence types, 109 AMR genes, and 53 plasmid groups [12]. The results revealed that sequence types (ST88, ST100) and serotypes (O9:H19, O116:H11, O149:H10) of *E. coli* isolates exhibited enhanced virulence, while F18ab-fimbriated isolates carrying stx2A genes showed higher cytotoxicity, 95.8% were multidrug resistant, and 79% harbored oqxB-aac(3) gene clusters [13].

These findings reflect global concerns where MDR Gram-negative pathogens contribute to 60–70% of antimicrobial treatment failures in veterinary practice, highlighting critical challenges for animal health management [14].

Extended-spectrum beta-lactamase (ESBL)-producing-*E. coli* is spreading worldwide and poses a public health issue [15]. *Escherichia coli* has evolved multiple β-lactamase-mediated resistance mechanisms, including extended-spectrum β-lactamases (ESBLs; SHV, TEM, OXA, and CTX-M types), plasmid-mediated AmpC β-lactamases (CMY-2, a plasmid-mediated *AmpC*-like gene) and carbapenemases (MBL, KPC, and class D oxacillinases) [16]. CTX-Ms formed the largest group of ESBLs, and they have become globally disseminated. Currently, CTX-M-15 (a part of the CTX-M-1 group) is the most frequent CTX-M worldwide. This is closely followed by CTX-M-14 (a member of the CTX-M-9 group) [17]. Given the limited data on the epidemiology of MDR *E. coli* from pigs in Hubei province in China, it is interesting to investigate the spread of these resistant bacteria, particularly the ESBL types, which may be the cause of the spread of resistance genes from pork to human.

Several epidemiological studies on *E. coli* have been initiated in parts of China. In northeastern China, a survey showed that the separation rate of *E. coli* isolated from pig fecal samples reached 88% [18]. An investigation on pig farms in Henan province found that the positive rate of *E. coli* was 70.74%, of which the commonly sequenced types (STs) 10 and 101 were identified [19]. However, data on the genetic diversity and antimicrobial resistance of *E. coli* is still restricted in the Hubei province of central China.

*E. coli* can induce porcine postweaning diarrhea (PWD) [20], and it may be associated with the expression of fimbriae to mediate adhesion and colonization of porcine enterocytes [21,22]. Most adhesins are related with fimbriae F18 and F4 (K88), F5 (K99), F6 (987P) and F41, with F18 and F4 (K88) being leading causes of PWD in piglets [23,24,25]. The predominant toxins of pathogenic *E. coli* include those that are heat-labile (LT-I and LT-II) [26,27] and heat-stable (STa and STb) [28], as well as enteroaggregative *E. coli* heat-stable enterotoxin 1 (EAST1) [29], and these produce other virulence factors, such as pathogenicity islands (PAI). The locus of enterocyte effacement (LEE) and high-pathogenicity islands (HPIs) are particularly significant PAIs. *Irp2* and *fyuA* genes, located in high-pathogenicity islands (HPIs), encode a yesiniabactin-like iron-scavenging system [30]. However, *E. coli* isolated from pigs with diarrhea carry different types of virulence genes, which can enhance the pathogenicity of *E. coli* through synergistic effects. However, data on characteristics of virulence genes of *E. coli* is still restricted in the Hubei province of central China.

Multilocus sequence typing (MLST) has been used to study the evolution and epidemiology of a number of bacterial pathogens. It has become the method of choice for typing epidemiologically important isolates. MLST is a widely used and standardized molecular typing method that enables comparison of *E. coli* lineages across studies and geographic regions, and is particularly suitable for population structure and epidemiological investigations [31]. Investigating the trend characterization of epidemic isolates will help us to better understand epidemiology.

This present study was carried out to investigate the prevalence and characteristics of *E. coli*. The MLST genotypes, antibiotic resistance and virulence genes of *E. coli* isolates were examined, having been collected from 2019 to 2022 in Hubei province of China. These findings provide information and implications for safeguarding and commanding the occurrence of diseases in future studies.

## 2. Results

### 2.1. Isolation of E. coli Isolates and Antimicrobial Susceptibility Profile

**Figure 1:**
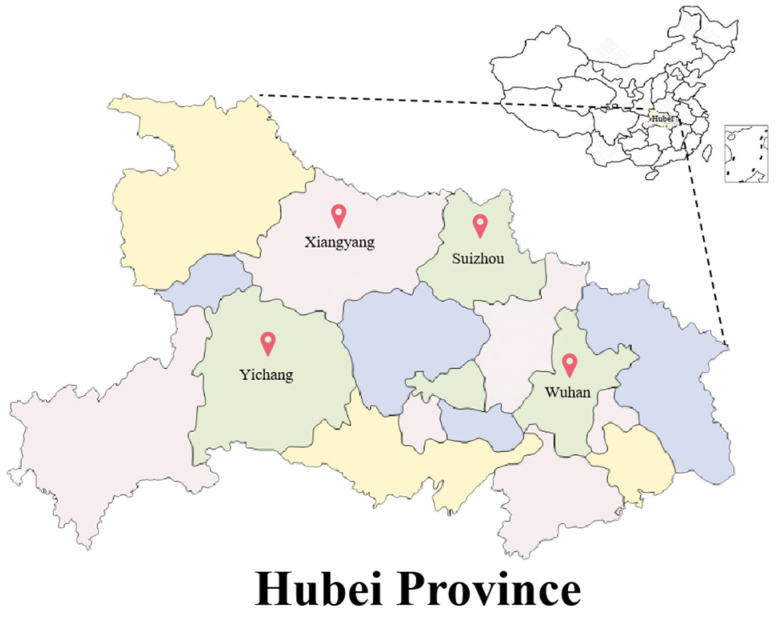
Geographical distribution of the farms where the experimental samples were collected. Farms represent the different farms where samples were collected and the number of samples; Wuhan (82), Xiangyang (37), Yichang (14), Suizhou (15).

*E. coli* isolates were obtained from the fecal matter of weaned piglets from Hubei province. A total of 148 isolates of *E. coli* were separated from samples and further identified by PCR tests as *E. coli*. There are 15 isolates of *E. coli* isolated from Suizhou city, 37 isolates of *E. coli* isolated from Xiangyang city, 82 isolates of *E. coli* isolated from Wuhan city and 14 isolates of *E. coli* isolated from Yichang city (Figure 1).

### 2.2. Antimicrobial Susceptibility Profile of E. coli Isolates

**Figure 2:**
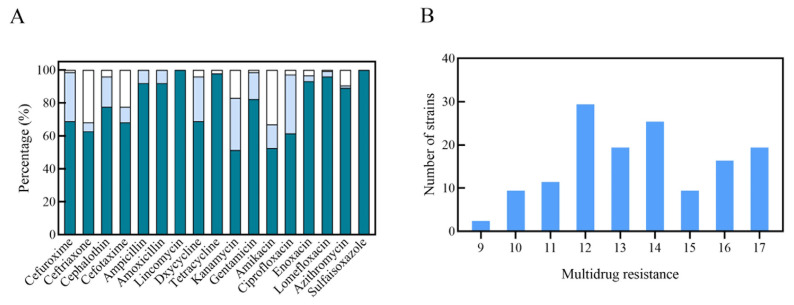
Antimicrobial susceptibility results of *E. coli* (*n* = 148) isolates. (**A**) Antimicrobial resistance profiles of *E. coli* isolates to 17 agents. (**B**) Analysis of multidrug resistance. X-axis indicates multidrug resistance of the isolates to 17 agents. (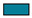 drug resistance rate; 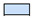 drug intermediation rate; 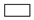 drug sensitivity rate).

**Figure 3:**
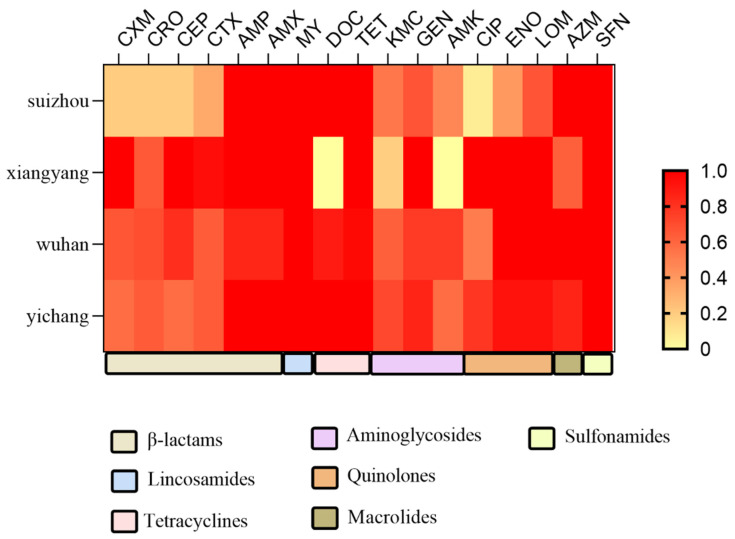
Heatmap showing the percentage of pig farm *E. coli* isolates resistant to each of the antibiotics tested. The resistance rate of *E. coli* isolates to 17 agents. CXM, cefuroxime; CRO, ceftriaxone; CEP, cephalothin; CTX, cefotaxime; AMP, ampicillin; AMX, amoxicillin; MY, lincomycin; DOC, doxycycline; TET, tetracycline; KMC, kanamycin; GEN, gentamicin; AMK, amikacin; CIP, ciprofloxacin; ENO, enoxacin; LOM, lomefloxacin; AZM, azithromycin; SFN, sulfaisoxazole.

Antimicrobial susceptibility testing (AST) results showed that all of the *E. coli* isolates showed resistance to lincosamides and sulfonamide (Figure 2A). A high rate of resistance to sulfaisoxazole, tetracycline, lomefloxacin, enoxacin, ampicillin, amoxicillin, azithromycin, and gentamicin has also been observed for the isolates, among which 100% of the *E. coli* was resistant to sulfaisoxazole; 97.97% of the *E. coli* was resistant to tetracycline; 95.95% of the *E. coli* was resistant to lomefloxacin; 93.24% of the *E. coli* was resistant to enoxacin; 91.89% of the *E. coli* was resistant to ampicillin; 91.89% of the *E. coli* was resistant to amoxicillin; 89.19% of the *E. coli* was resistant to azithromycin; and 82.43% of the *E. coli* was resistant to gentamicin. The isolates demonstrated a relatively low rate of resistance to cephalothin (77.7%) cefuroxime (68.92%), doxycycline (68.92%), cefotaxime (68.24%), ceftriaxone (62.84%), ciprofloxacin (61.49%), amikacin (52.7%) and kanamycin (51.35%) (Figure 2A and Figure 3).

As shown in Figure 2B, all of the isolates were multidrug-resistant (MDR) isolates, and most of the isolates were resistant to more than three drug classes. AST revealed that the 20 isolates showed severe resistance profiles that were resistant to the seventeen antimicrobial agents tested. Overall, most of the isolates were conferring resistance to 12–14 of the antimicrobial agents tested. Among the antibiotics tested, high resistance rates were observed for sulfaisoxazole, azithromycin, lomefloxacin, enoxacin, amikacin, tetracycline, doxycycline, lincomycin, cefuroxime, ceftriaxone, cephalothin, and cefotaxime.

*E. coli* in different regions has separate antibiotic resistance. The AST results revealed that resistance to lincomycin, and sulfaisoxazole was a common phenotype of the isolates from the pig farms in four cities, including Xiangyang, Yichang, Suizhou, and Wuhan in Hubei province (Figure 3). *E. coli* isolates resistant to doxycycline and amikacin were isolated, including in Yichang, Suizhou, and Wuhan in Hubei province; Xiangyang was the only region from which no isolates from pig farms with the abovementioned resistant phenotypes were detected (Figure 3). Notably, MDR isolates were identified on pig farms in Hubei province, but a relatively high proportion of the MDR isolates were identified on farms in Wuhan and Yichang relative to those from the other city.

### 2.3. Detection of Antimicrobial Resistance Genes

**Figure 4:**
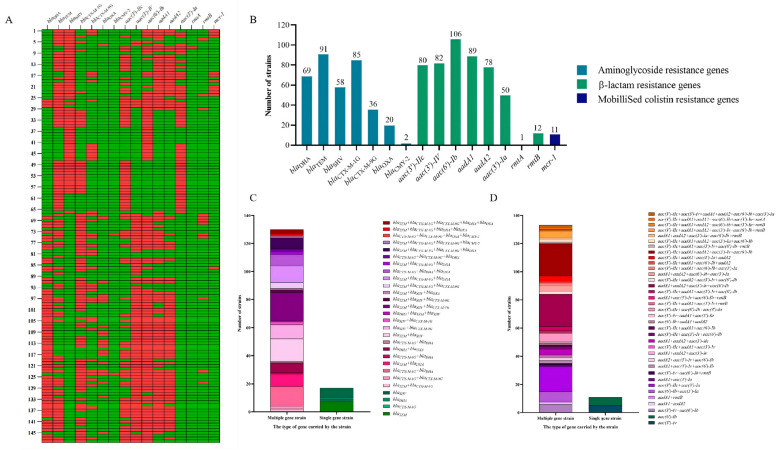
Detection rate of antibiotic resistance genes in *E. coli* isolates. (**A**) Heat map showing detected antimicrobial resistance genes carried by the isolates. Small rectangles marked in red and green represent positive and negative. (**B**) The bar graph shows the numbers of positive isolates for different detected antibiotic resistance genes. Antimicrobial resistance genes accounting for resistance to the same class of antibiotics are marked with bars in the same color. (**C**) Genetic compositions of extended-spectrum β-lactamase isolates. Left column: multiple gene isolates, right column: single gene isolates. (**D**) Genetic compositions of aminoglycoside-modifying enzyme isolates. Left column: multiple gene isolates, right column: single gene isolates.

16 antibiotic resistance genes (ARGs) in 148 isolates of *E. coli* were detected (Figure 4A) using PCR-based assays. Of these, *aac(6′)-Ib* (71.6%, 106/148), *bla*_TEM_ (61.5%, 91/148), *aadA1* (60.1%, 89/148), *bla*_CTX-M-1G_ (57.4%, 85/148), *aac(3′)-Iv* (55.4%, 82/148), *aac(3′)-IIc* (54.1%, 80/148), *aadA2* (52.7%, 78/148), and *bla*_DHA_ (46.9%, 69/148) were prevalent with higher detection rates (Figure 4B). Among the 148 *E. coli* isolates, 147 isolates carried β-lactamase genes with a detection rate of 99.3%, of which, the detection rates of *bla*_SHV_, *bla*_TEM_, *bla*_OXA_, *bla*_CMY-2_ and *bla*_DHA_ were 39.2% (58/148), 61.5% (91/148), 13.5% (20/148),1.4% (2/148) and 46.6% (69/148), respectively. The detection rate of *bla*_CTX-M-1G_ in CTX-M was 57.4% (highest) and the detection rate of *bla*_CTX-M-9G_ was 24.3%.

Among the 148 *E. coli* isolates, 144 isolates carried aminoglycoside-modifying enzyme genes with a detection rate of 97.3%, of which the detection rates of *rmtB* (8.1%) and *aac(3′)-Ia* (33.8%) were prevalent with lower detection rates. Amongst the isolates positive for aminoglycoside-modifying enzyme genes, only one strain was positive for *rmtA*. In addition, the detection rate of the mobilized colistin resistance gene *mcr-1* was 7.4%.

Antibiotic resistance genes were detected in all 147 isolates, with only one strain having no detected antibiotic genes. Of the 147 isolates, 91 (61.5%) had more than 6 ARGs, 4 (2.7%) isolates had more than 10 ARGs, and 2 isolates had 11 ARGs (Supplementary Table S1).

### 2.4. Various Extended-Spectrum β-Lactamase Genes Were Present in the Isolates

Among all of the isolates tested, 147 (99.3%) carried ESBLs and 130 of them harbored various number of ESBLs. The most frequent was *bla*_TEM_ + *bla*_SHV_ + *bla*_CTX-M-1G_ (13.5%, 20/148); 64 isolates were found to have two different ESBLs genes; 52 isolates were found to have three different ESBLs genes; 11 isolates were found to have four different ESBLs genes; and 3 isolates were found to have five ESBLs genes—*bla*_TEM_, *bla*_CTX-M-1G_, *bla*_CTX-M-9G_, *bla*_DHA_ and *bla*_OXA_ (Figure 4C, Supplementary Table S1).

### 2.5. Various Extended-Spectrum Aminoglycoside-Modifying Enzyme Genes Were Present in the Isolates

Amongst the 148 isolates tested, 144 (97.3%) isolates carried aminoglycoside-modifying enzyme genes and 133 of them harbored various numbers of aminoglycoside-modifying enzyme genes. The most frequent were *aadA1 + aadA2 + aac(3′)-Iv + aac(6′)-Ib* and *aac(3′)-IIc* + *aadA1* + *aadA2* + *aac(3′)-Iv* + *aac(6′)-Ib* (15.5%, 23/148); 34 isolates were found to have two different ESBLs genes; 22 isolates were found to have three different ESBLs genes; 41 isolates were found to have four different ESBLs genes; 27 isolates were found to have five different ESBLs genes; and 9 isolates were found to have six different ESBLs genes (Figure 4D, Supplementary Table S1).

### 2.6. Prevalence of Virulence Genes in E. coli Isolates from Pigs

**Figure 5:**
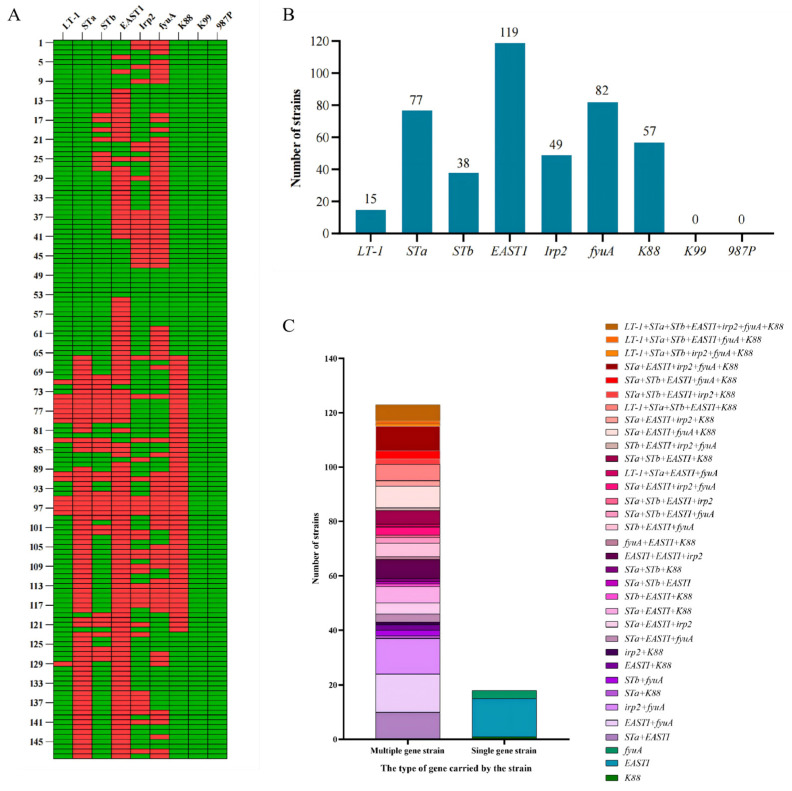
Distribution and category of *E. coli* isolates positive for virulence-associated genes. (**A**) Heat map showing detected virulence genes carried by the isolates. Small rectangles marked in red and green represent positive and negative. (**B**) The bar graph showing numbers of positive isolates for different detected virulence genes. (**C**) Genetic compositions of virulence-associated isolates. Left column: multiple gene isolates, right column: single gene isolates.

The distribution of eight virulence genes in 148 isolates of *E. coli* has been examined in this study. As shown in Figure 5A,B, the virulence gene *EASTI* (80.4%, 119/148) was found in most of the *E. coli* isolates, followed by *fyuA* (55.4%, 82/148), *STa* (52%, 77/148), *K88* (38.5%, 57/148) and *irp2* (3.1%, 49/148). While *K99* and *987P* were not detected in all isolates.

Among the 148 isolates tested, 141 (95.3%) carried virulence genes and 123 of these harbored a various number of virulence genes. The most frequent were *EASTI+* and *EASTI* + *fyuA*+ (9.9%, 14/141), followed by *irp2* + *fyuA*+ (9.2%, 13/141), *STa* + *EASTI* (7.1%, 10/141), *STa* + *EASTI* + *irp2* + *fyuA* + *K88*+ (6.4%, 9/141), *STa* + *EASTI* + *fyuA* + *K88*+ (5.7%, 8/141), *EASTI* + *EASTI* + *irp2*+ (5.0%, 7/141), *LT-1* + *STa* + *STb* + *EASTI* + *irp2* + *fyuA* + *K88*+ (4.3%, 6/141), *LT-1* + *STa* + *STb* + *EASTI* + *K88*+ (4.3%, 6/141), and *STa* + *EASTI + K88*+ (4.3%, 6/141) (Figure 5C, Supplementary Table S1).

### 2.7. Multi-Locus Sequence Typing (MLST)

**Figure 6:**
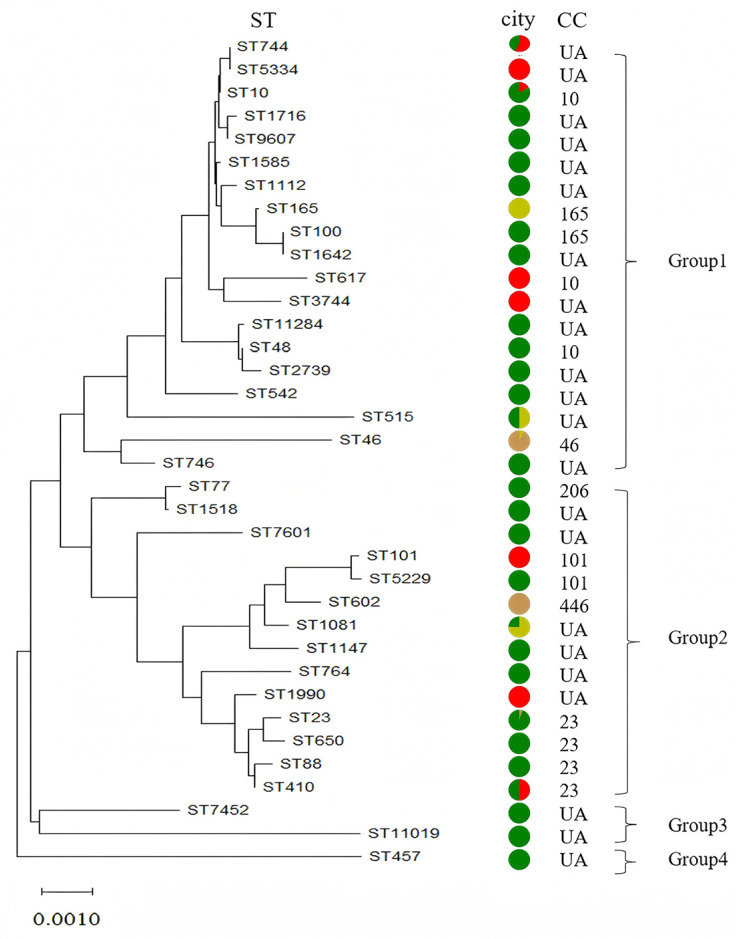
Neighbor-joining phylogenetic tree of *E. coli* isolates based on concatenated MLST sequences of seven housekeeping genes. Branch lengths represent genetic distances calculated using the Kimura two-parameter model. [color code (isolate origin city): green = Wuhan; red = Yichang; brown = Xiangyang; and yellow = Suizhou. Abbreviations: ST = sequence type; CC = clone complex; UA = unassigned clone complex. The dendrogram is clustered into four groups (Group 1–4) based on MLST profile similarity].

The genetic diversity of these *E. coli* isolates was analyzed with MLST. A total of 148 isolates were analyzed utilizing MLST, the identification of 38 sequence types (STs). Among these isolates, 101 out of the 148 isolates possessed 14 different STs which belonged to 7 CCs. The remaining 46 isolates belonged to 23 different unassigned STs. The presence of new STs was due to new combinations of previously known alleles in *adk* (allele 7), *fumC* (allele 7), *gyrB* (allele 193), *icd* (allele 1), *mdh* (allele 986), *purA* (allele 159) and *recA* (allele 139). The predominant STs were ST46, ST23 and ST10 containing 29 (19.59%), 20 (13.51%), 12 (8.11%) isolates respectively. Ten STs contained three or more isolates with ST602, ST165, ST100, ST744, ST7452, ST1642, ST1081, ST515, ST48 and ST101 comprising nine (6.08%), eight (5.41%), eight (5,41%), seven (4.73%), five (3.38%), four (2.70%), four (2.70%), four (2.70%), three (2.03%) and three (2.03%) isolates, respectively. Seven STs (ST410, ST650, ST764, ST1147, ST1518, ST2739 and ST5229) contained two isolates each. Eight STs (ST77, ST88, ST457, ST542, ST617, ST710, ST746, ST1112, ST1585, ST1716, ST1990, ST3744, ST5334, ST7601, ST9607, ST11019 and ST11284) had only one isolate each (Figure 6).

Minimum-spanning trees showed that the tested *E. coli* were mainly classed into seven clonal complexes and other unassigned clone complexes. CC-46 was the most frequently isolated clonal complex, containing 29 isolates belonging to one ST, and accounted for 19.59% (29/148) of all the isolates. The major clonal complexes also included CC-23 (25/148, 16.89%), CC-10 (16/148, 10.81%) and CC-165 (16/148, 10.81%). The other isolates covered 28 STs belonging to three CCs and were unassigned.

To further analyze sequence types (STs), a neighbor-joining phylogenetic analysis was performed based on concatenated MLST sequences (Figure 6). The NJ phylogram classified the identified STs into four major phylogenetic groups. Group 4 comprised a single isolate belonging to an unassigned ST, whereas Group 3 included six isolates representing two different unassigned STs. In contrast, Groups 1 and 2 encompassed the majority of isolates (*n* = 140) and contained most of the dominant clonal complexes, including CC-10, CC-23, CC-46, CC-101, CC-165, CC-206, CC-446, as well as several unassigned STs. Specifically, CC-10 included ST10, ST48, and ST617; CC-23 included ST23, ST88, ST410, and ST650; CC-101 included ST101 and ST5229; and CC-165 included ST100 and ST165 (Figure 6).

## 3. Discussion

*E. coli* is one of the main pathogenic bacteria that impact the production and growth of pigs on pig farms. It is associated with gastrointestinal diseases such as diarrhea, edema disease, and systemic infections such as septicemia and polyserositis [2]. These diseases caused by *E. coli* can increase mortality, morbidity and growth delays of piglets, which are responsible for economic losses. This study analyzed the prevalence, genetic diversity and antibiotic resistance of disease, which may help us to improve methods of prevention and treatment.

*E. coli* isolates exhibit high AMR with widespread multidrug resistance and extensive carriage of resistance genes, showing regional differences in resistance phenotypes.

From 2002 to 2008, the prevalence of *E. coli* isolated from pork chop samples was 44% in the United States [32]. However, various incidence rates have also been reported in different regions of China. From 2003 to 2009, the prevalence of *E. coli* isolates from pig farms was 77.78% in China [33]. From 2013 to 2016, the positive rates of *E. coli* between farm 1 and farm 2 were 40.25% and 59.75%. respectively, in Guangdong province [11]. Between 2016 and 2017, a survey indicated that the separation rate of *E. coli* isolated from pig fecal swabs reached 88% in northeastern China, including Heilongjiang, Jilin and Liaoning [18].

Diarrhea in weaned piglets driven by *E. coli* remains a principal cause of economic losses for the pig industry. This commonly requires antimicrobial drug treatment, which is of considerable expense when used to cure piglets infected with this pathogen. In 2011, the study of Agersө et al. found that 32% of isolates have multi-drug resistance, mainly concentrated on ampicillin (27%) and tetracycline (29%) [34]. In 2012, Tadesse et al. tested 1729 isolates of *E. coli* antibiotic susceptibility varied from different sources, the resistance rate of *E. coli* increased from 7.2% to 63.6% but the most common resistance was to tetracycline, streptomycin and sulfonamides [35]. A total of 131 *E. coli* isolates were obtained from the pigs presenting from diarrhea in Switzerland from 2014 to 2015, the isolates exhibited resistance to tetracycline (50%), sulfamethoxazole (49%), ampicillin (26%), gentamicin (17%), and ciprofloxacin (8%) [36]. However, this caused a rise in the employment of various antimicrobial agents, such as lincosamides, tetracyclines and sulfonamides, which may expand antimicrobial resistance.

In this study, antimicrobial susceptibility testing of the *E. coli* isolates revealed the highest prevalence of resistance to three classes of antimicrobials: lincosamides, tetracyclines, and sulfonamides. This observation is likely associated with the long-term irrational use of these three antibiotics for controlling bacterial diarrhea in piglets within Hubei province.

Notably, over 80% of the *E. coli* isolates exhibited high resistance rates to seven antimicrobials, specifically tetracycline, lomefloxacin, enoxacin, ampicillin, amoxicillin, azithromycin, and gentamicin. Resistance was also detected against β-lactam drugs: the resistance rate to cephalothin (a first-generation cephalosporin) reached 77.7%, which was higher than that to cefuroxime (a second-generation cephalosporin, 68.9%), the third-generation cephalosporins cefotaxime (68.2%) and ceftriaxone (62.8%).

All isolates displayed varying degrees of resistance to the tested antimicrobials, and most *E. coli* isolates exhibited a high prevalence of multidrug resistance. Among them, 20 isolates were resistant to all 17 tested antimicrobials. Overall, more than half of the isolates showed resistance to 9–17 antimicrobial agents. The most common MDR pattern was resistance to 12 antimicrobials (cefuroxime, amikacin, ampicillin, amoxicillin, lincomycin, doxycycline, tetracycline, gentamicin, enoxacin, lomefloxacin, azithromycin, and sulfaisoxazole), which was observed in 30 isolates.

For context, relevant studies conducted in other regions of China have reported comparable trends. Jiang et al. found that *E. coli* isolates had high resistance rates to ampicillin (99.5%), tetracycline (93.4%), and amoxicillin (65.1%), with prevalent resistance also detected against cephalosporins, quinolones, and aminoglycosides [37]. Meng et al. similarly noted that the majority of *E. coli* isolates were resistant to tetracycline (79.57%), trimethoprim-sulfamethoxazole (73.12%), and kanamycin (55.91%) [10]. In contrast, a study in Sichuan province (2012–2013) reported that *E. coli* isolates had the highest resistance to sulfamethoxazole (61.6%), followed by tetracycline (61.2%), ampicillin (48.2%), and kanamycin (22.4%) [38]. Additionally, a multi-provincial survey (covering seven provinces) showed that *E. coli* isolates from pig farms had resistance rates of 81.44% to ampicillin, 94.37% to tetracycline, and 88.36% to sulfaisoxazole [39]. Collectively, these findings provide critical insights and implications for guiding the rational application of antibiotics in swine production in future research and practice.

It has been documented that *E. coli* has a great ability to accumulate ARGs, mainly through horizontal gene transfer [40]. In this study, a large number of ARGs are detected in various isolates and it was found that a higher proportion contained six or more resistance genes. One of the most common antibiotic resistance mechanisms in *E. coli* is mediated by the production of β-lactamase, ESBLs are an increasing cause of resistance to third-generation cephalosporins, including the fourth-generation cephalosporins in Enterobacteriaceae [41,42]. The *bla*_TEM_, *bla*_CTX-M_ and *bla*_SHV_ types have been recognized as the most prevalent ESBL genes that confer antibiotic resistance among pathogens [43,44]. In this study, five ESBL genes and two AmpC enzyme genes (*bla*_DHA_, *bla*_CMY-2_) were detected. Among the five ESBL genes, *bla*_TEM_ (61.5%) was the most prevalent gene, similar to the ESBL epidemic in Guizhou in 2021 [45]; followed by *bla*_CTX-M-1_ at 57.4%, *bla*_SHV_ at 39.2%, *bla*_CTX-M-9G_ at 24.3% and *bla*_OXA_ at 13.5%. ESBLs are paradigmatic of resistance, are usually encoded on mobile genetic elements that accelerate their dissemination, and have become another challenge for drug resistance control in pig farms [46].

It is inevitable for bacteria to develop resistance to aminoglycosides due to their widespread use. There have been reports of aminoglycoside resistance in both Gram-negative and Gram-positive bacteria [47]. In this study, the detection rate of five aminoglycoside-modifying enzyme genes (*aac(6′)-Ib*, *aac(3′)-IV*, *aac(3′)-IIc*, *aadA1*, *aadA2*) exceeds 50%, and it is possible to carry multiple aminoglycoside modifying enzyme genes in a single strain, leading to high levels of resistance to aminoglycosides. In 2015, the plasmid-mediated colistin resistance gene, *mcr-1*, was reported for the first time in *E. coli* isolate from the animals and their food in China [48], it has quickly spread to human pathogens [49]. The transfer of colistin resistance by plasmid has been ascribed to the *mcr-1* gene, which is the most predominant type of *mcr* gene [50]. The low detection rate (7.4%) of *mcr-1* in this study likely results from the low use of polymyxins in pig feed in Hubei. It is important to monitor such isolates closely to prevent their spread. The concern of antibiotic resistance in these categories is serious and needs urgent attention. Additionally, it demands urgent attention in terms of the regularization monitoring of antimicrobial susceptibilities and the efficient administration of bacterial infections to restrict the further spread of multidrug resistance in Hubei province.

In past decades, the occurrence and spread of PWD in piglets has caused massive economic losses to the development of the pig farming industry in China [51]. The highest detection rate of virulence factor has been found for *EAST1* (80.4%), followed by *fyuA* (55.4%). Fimbriae adhesins are necessary in the pathogenetic mechanism, the most common adhesins on *E. coli* from PWD in pigs are fimbriae *F4* and *F18* [52]. *F4* (*K88*) (38.5%) was identified in this study, which indicates that it is closely related with pathogenic *E. coli.* For enterotoxins detected in this study, the gene encoding the heat-stable enterotoxin *STa* was frequently detected (52.0%), which is similar to findings reported in Korea and other countries [53,54], followed by the heat-stable enterotoxin *STb* (25.7%). The detection of heat-labile enterotoxin (*LT-1*) (10.1%) was less than that of the *STa* and *STb* in this study. It is possible that the comparative identification of enterotoxins seems to vary from one geographic area to another. This study finds the distribution and characteristics of virulence factors in *E. coli* in Hubei province, and the data may be useful for establishing preventive measures for PWD.

The application of MLST in *E. coli* isolates facilitates a better understanding of the genetic diversity of these *E. coli* isolates. In this study, a total of 148 isolates were analyzed utilizing MLST, leading to the identification of 38 sequence types (STs), the most frequent ST was ST46, followed by ST23, ST10, ST602, ST165, ST100 and ST744. These isolates belonged to seven CCs and other unassigned clone complexes, including CC46, CC23, CC10 and so on. This reveals that there is abundant diversity in ST types among porcine *E. coli* isolates in Hubei province. Notably, the ST distribution profile in our study exhibits distinct discrepancies compared with previously reported data, both from the same region (Hubei province) and other regions of China. First, from the city-level perspective, no city-exclusive STs were identified in this study: dominant sequence types (e.g., ST46, ST23, ST10) were detected across multiple cities in Hubei province, indicating regional circulation of these lineages rather than strict geographic restriction. This pattern may reflect potential dissemination via animal movement, shared farming practices, or environmental transmission routes between farms in different cities. Second, our study identified ST46 as the most prevalent sequence type, which differs from findings reported in other regions of China. For instance, Yang et al. [55] found that ST10 was the most common sequence type (22/171, 12.9%), followed by ST48 (16/171, 9.4%) and ST744 (8/171, 4.7%). Additionally, the prevalence of ST10 in our study (12/148, 8.11%) was lower than that documented in previous research. Zhang et al. [11] have reported that the proportion of ST10 reached 50% (16/32). The potential reasons are the limitation of the data size, regional epidemiological characteristics of *E. coli* clones, and differences in sample collection strategies (e.g., variations in farm scale, feeding patterns, and disease prevalence periods between studies). Recently, *E. coli* ST10 has not only been detected in animals in China but also isolated from human infections in China [56], indicating the potential zoonotic risk of this ST clone, which warrants further surveillance. These findings highlight the necessity of the continuous regional epidemiological surveillance of *E. coli* ST types, as the distribution of dominant clones may vary geographically, which is crucial for guiding local disease prevention and control strategies.

Based on the detected virulence gene combinations, most isolates could be classified as enterotoxigenic *E. coli* (ETEC), which is consistent with their isolation from diarrheic piglets. Several dominant STs, including ST46, ST23, and ST10, were detected across multiple cities, suggesting regional dissemination rather than city-specific circulation. Notably, some of these STs have also been reported in human infections, indicating potential zoonotic relevance. Although this study was not designed to establish causal relationships between virulence and antimicrobial resistance, an isolate-level integrative analysis revealed that several multidrug-resistant isolates simultaneously harbored multiple virulence-associated genes. However, no clear linear correlation was observed between the number of resistance genes and virulence genes across isolates. This finding suggests that antimicrobial resistance and virulence traits are not directly proportional but may be co-selected under similar selective pressures in intensive swine production systems.

Dominant STs exhibited heterogeneous resistance and virulence profiles, indicating that clonal background alone does not determine pathogenic potential. The coexistence of multidrug resistance and enterotoxigenic virulence factors in several isolates underscores their epidemiological relevance. The detection of similar STs across multiple cities supports the hypothesis of regional dissemination and highlights the importance of continuous surveillance to monitor the spread of multidrug-resistant *E. coli* within and beyond swine populations.

Several limitations should be acknowledged in this study. First, although phenotypic resistance to sulfonamides was universally observed, sulfonamide resistance genes were not investigated, which limits mechanistic interpretation and warrants further molecular characterization in future studies. Second, while multiple antimicrobial resistance genes and virulence-associated genes were detected, a systematic one-to-one correlation between resistance genotype and phenotypic susceptibility, as well as between virulence gene content and clinical severity, could not be established. This was partly due to the absence of detailed clinical outcome data and individual antimicrobial treatment histories for the sampled piglets. Third, although transmission patterns and horizontal gene transfer were discussed, the lack of whole-genome sequencing data constrained comprehensive analyses of mobile genetic elements and resistance gene dissemination. Future studies integrating genomic, clinical, and antimicrobial usage data will be essential to identify high-risk lineages and better understand the evolution and spread of multidrug-resistant *E. coli*.

In this study, a high antimicrobial resistance and the genotypic diversity of *E. coli* were isolated from swine in Hubei province and observed. From the results obtained it can be concluded that these isolates present highly prevalent multi-drug resistance. These data provide a greater understanding of the genetic diversity and antimicrobial resistance of *E. coli*. From a One Health perspective, the “risk” associated with individual isolates should be interpreted as a composite characteristic arising from the combined presence of multidrug resistance and key virulence determinants rather than from sequence type alone. The presence of multidrug-resistant diarrheagenic *E. coli* in swine populations represents a potential reservoir for resistance determinants that may disseminate across animals, humans, and the environment, emphasizing the need for integrated antimicrobial stewardship strategies.

## 4. Materials and Methods

### 4.1. Bacterial Isolates and Identification

From 2019 to 2022, a total of 148 fecal swab samples were collected from pig farms in 4 cities: Xiangyang, Yichang, Suizhou, and Wuhan in Hubei province. The samples were taken from diseased piglets with diarrhea symptoms. These isolates were obtained using MacConkey agar, which was incubated at 37 °C for 24 h. Pink colonies were picked and purified again, then bacterial isolates cultured in Luria–Bertani (LB) broth at 37 °C for 24 h. And further confirmed by the PCR amplification of the 16S rRNA gene with primers (16S-F: 5′-AGAGTTTGATCATGGCTCAG-3′; 16S-R: 5′-TAGGGTTACCTTGTTACGACTT-3′) as previously described [55]. PCR amplification was performed as previously described, with minor modifications to the annealing temperature and extension time to optimize amplification efficiency. Primer sequences remained unchanged.

### 4.2. Antibiotic Resistance Profiles

According to the guidelines of the Clinical and Laboratory Standards Institute [57], the confirmed *E. coli* were assayed for antimicrobial susceptibility testing. *E. coli* isolates were examined for susceptibility to antimicrobial drugs utilizing a disk diffusion assay. All samples were analyzed for the presence of resistant bacteria. A total of 17 antimicrobials were tested, comprising cefuroxime (CXM), ceftriaxone (CRO), cephalothin (CEP), cefotaxime (CTX), ampicillin (AMP), amoxicillin (AMX), lincomycin (MY), doxycycline (DOC), tetracycline (TET), kanamycin (KMC), gentamicin (GEN), amikacin (AMK), ciprofloxacin (CIP), enoxacin (ENO), lomefloxacin (LOM), azithromycin (AZM), and sulfaisoxazole (SFN). Inoculated plates were incubated at 37 °C for 24 h and the diameters of the inhibition zone were subsequently measured (in mm). Antimicrobial susceptibility testing was performed using the disk diffusion method. Isolates were categorized as susceptible, intermediate, or resistant according to the Clinical and Laboratory Standards Institute (CLSI) M100 guidelines based on inhibition zone diameters. The *E. coli* strain ATCC 25922 was utilized for quality control. Multidrug resistance (MDR) was defined as resistance to at least one antimicrobial agent in three or more different antimicrobial classes, in accordance with established criteria [58]. The antimicrobial agents tested in this study represented eight antimicrobial classes, including cephalosporins, penicillins, lincosamides, tetracyclines, aminoglycosides, fluoroquinolones, macrolides, and sulfonamides.

### 4.3. Detection of Antibiotic Resistance Genes

To extract the total DNA of each *E. coli* strain, we boiled the lysis of the isolated colonies. PCR was used to identify genes responsible for resistance to β-lactamase genes (*bla*_DHA_, *bla*_CMY-2_, *bla*_TEM_, *bla*_SHV_, *bla*_CTX-M-1G_, *bla*_CTX-M-9G_, *bla*_OXA_), aminoglycoside-modifying enzyme genes (*aac(3′)-Ia*, *aac(3′)-IIc*, *aac(3′)-IV*, *aac(6′)-Ib*, *aadA1*, *aadA2*, *rmtA*, *rmtB*), and polypeptide resistance gene mcr-1. The primers for PCR are listed in Table 1 and were synthesized at Sangon Biotech, Shanghai, China.

### 4.4. Detection of Virulence-Associated Genes

Considering the contribution of virulence genes to the invasiveness and pathogenicity of *E. coli*, DNA was isolated and we identified nine virulence-associated genes of each strain, including *LT-1*, *STa*, *STb*, *EAST1*, *irp2*, *fyuA*, *K88*, *K99*, and *987P*, which were amplified by PCR, as previously described [51,68,69,70]. These virulence genes were selected because they represent major adhesins, enterotoxins, and pathogenicity island-associated factors commonly implicated in porcine enterotoxigenic *E. coli* (ETEC) infections. The primer sequences of virulence genes are shown in Table 2. PCR reactions were carried out in a final volume of 25 μL containing 12.5 μL mix (Vazyme Biotech, Nanjing, China), 1 μL each of primers, and 2 μL bacterial DNA. PCR was performed in a GeneAmp PCR System 9700 (Applied Bio-systems, Darmstadt, Germany), under the following conditions: initial denaturation at 95 °C for 5 min; cycling consisted of 35 cycles of 30 s at 94 °C, 30 s at 58 °C, and 1 min at 72 °C, with a final extension at 72 °C for 10 min. The resulting amplification products were separated by electrophoresis in 1% agarose gel, stained with ethidium bromide and visualized using a GelDoc XR system (Bio-Rad, Shanghai, China).

### 4.5. MLST and Phylogenetic Tree

The multilocus sequence typing (MLST) was executed on *E. coli* isolates according to the *E. coli* MLST database guidelines (https://enterobase.warwick.ac.uk/species/ecoli/allele_st_search, accessed on 9 January 2023), according to the protocols published on the web site. Briefly, the seven house-keeping genes *adk*, *fumC*, *gyrB*, *icd*, *mdh*, *purA* and *recA* were amplified employing a PCR protocol, and the amplicons sequenced utilizing the amplification primers. We investigated individual gene sequences and allocated an allelic profile number in line with the MLST database. Sequence type (ST) and clone complex (CC) designations of each strain were composed of seven alleles. Multilocus sequence typing (MLST)-based phylogenetic analysis was performed using concatenated sequences of seven housekeeping genes. Sequence alignment was conducted in MEGA version 11.0, and a neighbor-joining (NJ) phylogenetic tree was constructed using the Kimura 2-parameter (K2P) substitution model. The robustness of the tree topology was assessed by 1000 bootstrap replicates.

### 4.6. Statistical Analysis

The Student’s *t*-test was employed to analyze the data. When *p* < 0.05, difference was considered statistically significant. The analysis was performed using GraphPad Prism version 8 (GraphPad Software, Inc., San Diego, CA, USA).

## Figures and Tables

**Table 1:** Primers used in this study to amplify *E. coli* drug resistance genes.

Genes	Primer Sequence (5′-3′)	Size of Product (Base Pairs)	Reference
*bla*_DHA_	F: AACTTTCACAGGTGTGCTGT R: CCGTACGCATACTGGCTTTC	387	Pai, Seo and Choi, 2007 [59]
*bla*_CMY-2_	F: ATGATGAAAAAATCGTTATGC R: TTGCAGCTTTTCAAGAATGCG	1143	Yan et al. 2004 [60]
*bla*_TEM_	F: ATAAAATTCTTGAAGACGAAA R: GACAGTTACCAATGCTTAATC	1080	Weill et al. 2004 [61]
*bla*_SHV_	F: CACTCAAGGATGTATTGTG R: TTAGCGTTGCCAGTGCTCG	885	Brinas et al. 2005 [62]
*bla*_CTX-M-1G_	F: CTTCCAGAATAAGGAATCCC R: CGTCTAAGGCGATAAACAAA	949	Liu et al. 2007 [63]
*bla*_CTX-M-9G_	F: TGACCGTATTGGGAGTTTG R: ACCAGTTACAGCCCTTCG	902	Liu et al. 2007 [63]
*bla*_OXA_	F: ATATCTCTACTGTTGCATCTCC R: AAACCCTTCAAACCATCC	619	Colom et al. 2003 [64]
*aac(3′)-Ia*	F: TTACGCAGCAGCAACGATGT R: GTTGGCCTCATGCTTGAGGA	402	Sun et al. 2012 [65]
*aac(3′)-IIc*	F: AACCGGTGACCTATTGATGG R: TGTGCTGGCACGATCGGAGT	774	Sun et al. 2012 [65]
*aac(3′)-IV*	F: GGCCACTTGGACTGATCGAG R: GCGGATGCAGGAAGATCAAC	609	Sun et al. 2012 [65]
*aac(6′)-Ib*	F: TTGCGATGCTCTATGAGTGGCTA R: CTCGAATGCCTGGCGTGTTT	482	Park et al. 2006 [66]
*aadA1*	F: AGGTAGTTGGCGTCATCGAG R: CAGTCGGCAGCGACATCCTT	589	Sun et al. 2012 [65]
*aadA2*	F: GGTGCTAAGCGTCATTGAGC R: GCTTCAAGGTTTCCCTCAGC	470	Sun et al. 2012 [65]
*rmtA*	F: CTAGCGTCCATCCTTTCCTC R: TTGCTTCCATGCCCTTGCC	635	Chen et al. 2004 [67]
*rmtB*	F: ACATCAACGATGCCCTCAC R: AAGTTCTGTTCCGATGGTC	724	Chen et al. 2004 [67]
*mcr-1*	F: CGGTCAGTCCGTTTGTTC R: CTTGGTCGGTCTGTAGGG	309	Liu et al. 2016 [48]

**Table 2:** Primers used in this study to amplify *E. coli* virulence-associated genes.

Virulence Factors	Primer Sequence (5′-3′)	Size of Product (Base Pairs)
LT-1	F: TAGAGACCGGTATTACAGAAATCTGA	282
	R: TCATCCCGAATTCTGTTATATATGTC	
STa	F: GGGTTGGCAATTTTTATTTCTGTA	183
	R: ATTACAACAAAGTTCACAGCAGTA	
STb	F: ATGTAAATACCTACAACGGGTGAT	300
	R: TATTTGGGCGCCAAAGCATGCTCC	
EAST1	F: ATGCCATCAACACAGTATATC	117
	R: TCAGGTCGCGAGTGACGG	
*irp2*	F: AAGGATTCGCTGTTACCGGAC	301
	R: TCGTCGGGCA GCGTTTCTTCT	
*fyuA*	F: TGATTAACCCCGCGACGGGAA	787
	R: CGCAGTAGGCACGATGTTGTA	
K88	F: GATGAAAAAGACTCTGATTGCA	841
	R: GATTGCTACGTTCAGCGGAGCG	
K99	F: CTGAAAAAAACACTGCTAGCTATT	543
	R: CATATAAGTGACTAAGAAGGATGC	
987P	F: GTTACTGCCAGTCTATGCCAAGTG	463
	R: TCGGTGTACCTGCTGAACGAATAG	

## Data Availability

The data presented in this study are available on request from the corresponding author.

## Supplementary Materials

The following supporting information can be downloaded at: https://www.mdpi.com/article/10.3390/ijms27010524/s1.
